# A systematic review and meta-analysis of health-related quality of life among patients with cardiovascular diseases in Ethiopia

**DOI:** 10.3389/fcvm.2024.1419538

**Published:** 2024-12-23

**Authors:** Worku Chekol Tassew, Agerie Mengistie Zeleke, Samson Sisay Woldie, Yeshiwas Ayale Ferede

**Affiliations:** ^1^ Medical Nursing Department, Teda Health Science College, Gondar, Ethiopia; ^2^ Clinical Midwifery Department, Teda Health Science College, Gondar, Ethiopia; ^3^ Department of Reproductive Health, Teda Health Science College, Gondar, Ethiopia

**Keywords:** health-related quality of life, cardiovascular disease, meta-analysis, systematic review, Ethiopia

## Abstract

**Introduction:**

Despite Ethiopia's best efforts, the physical, psychological, social, and environmental aspects of quality of life among patients with cardiovascular illnesses such as hypertension have not received adequate consideration. The quality of life among patients with cardiovascular diseases in Ethiopia has not been thoroughly examined; therefore, this study aimed to assess the prevalence and factors associated with health-related quality of life among patients with cardiovascular diseases.

**Methods:**

The results of this systematic review and meta-analysis were reported in accordance with the International Recommended Reporting items for Systematic Review and Meta-analysis guidelines. A thorough search of published literature was conducted utilizing reliable databases (PubMed) and web-based search platforms (Science Direct, African Journals Online, and Google Scholar). The extracted data were imported to STATA version 11 to determine the pooled prevalence of health-related quality of life. The heterogeneity among the results of the primary studies was analyzed using Cochran's *Q* test and quantified using *I*^2^ statistics. A funnel plot and Egger's test were used to determine the presence of publication bias.

**Results:**

This systematic review and meta-analysis included 10 published articles. The pooled prevalence of health-related quality of life among patients with cardiovascular diseases in Ethiopia was 45.32% [95% confidence interval (CI): 37.44–53.20, *P* < 0.001]. Age older than 60 years [odds ratio (OR) = 3.71, 95% CI: 2.81–4.89], presence of chronic comorbidities (OR: 2.87, 95% CI: 1.72–3.4.79), and rural residence (OR = 15.31, 95% CI: 2.82–83.26) were associated with poor health-related quality of life.

**Conclusion:**

According to the findings of this study, a large number of patients with cardiovascular diseases in Ethiopia experience poor health-related quality of life. Furthermore, age above 60 years, presence of comorbidities, and rural residence were found to have substantial impacts on the quality of life of patients. As a result, this review recommends that quality-of-life evaluation be included in routine patient treatment regimens.

**Systematic Review Registration:**

https://www.crd.york.ac.uk/prospero/display_record.php?RecordID=573993, identifier (CRD42024573993).

## Introduction

Cardiovascular disorders (CVDs) are a class of diseases that can harm the heart and/or blood vessels (1, 2). They are the main cause of morbidity and mortality globally, exerting catastrophic effects on individuals, families, healthcare providers, and the already overburdened healthcare system tied to health and the economy (1). According to the American Heart Association, approximately 19 million people are estimated to die from CVDs by 2022, with the majority of these deaths occurring in sub-Saharan nations (3). According to previous studies, the pooled prevalence of CVDs in Ethiopia is 5%, which contributes to substantial healthcare-related expenses (4). Because of the chronic nature of the disease and its complex long-term treatment, CVDs generate infinite consequences for patients’ health-related quality of life (HRQoL) (5).

The World Health Organization (WHO) defines health-related quality of life as a patient's perception of their place in life within the context of their culture and value systems, as well as their objectives, standards, expectations, and concerns. HRQoL emphasizes aspects of life that are influenced by social, psychological, environmental, and physical factors. A patient's perceived quality of life, which is related to their health, reflects their level of contentment with life conditions influenced by cardiovascular diseases (6). Factors such as patient's level of independence, psychological condition, physical health, social contact, and attitude all influence their HRQoL (7).

Health-related quality of life is a patient-reported outcome measure that determines how sickness, complications, and treatment affect a patient's health status. It can predict long-term medication adherence and persistence (8). Health-related quality of life is an important metric for assessing the effectiveness of health interventions and a useful component for predicting health outcomes in financial analysis (9). In low-income countries, treatment satisfaction and HRQoL can be negatively impacted by lower socioeconomic status, limited access, and expensive prescription costs (10). Poor HRQoL has a significant negative impact on both patients and medical institutions. In healthcare facilities, poor health-related quality of life increases the need for personnel, infrastructure, and patient flow. In addition, hospitalization, reduced income, strained social relationships, psychiatric problems, and physical disabilities all burdened up individuals with low HRQoL (11).

A long duration of disease, low educational status, the presence of cardiovascular disease symptoms, uncontrolled blood pressure, advanced age, poor treatment adherence, comorbidities, complications, multiple symptoms, a history of a stroke, and visual impairment are some of the contributing factors to poor health-related quality of life (12, 13). Furthermore, identifying indicators of poor health-related quality of life is critical for improving clinical care and determining intervention targets for illness prevention and treatment. Furthermore, assessing an individual’s health condition has been proven to be a more accurate predictor of mortality and morbidity than a variety of objective health measures (14).

In addition to cardiovascular medication, patient-reported problems need to be addressed to improve patient outcomes. Prior research has demonstrated that patients’ active participation in disease management can significantly improve HRQoL (15). Several general and disease-specific measures have been developed to assess the HRQoL of CVD patients. The European Quality of Life Five-Dimension-5-Level Scale (EQ-5D-5L) questionnaire is a widely used generic, preference-based, multi-attribute utility tool for assessing health technology in many countries and estimating the global disease burden in clinical practice. The tool measures patients’ health by creating a single summary utility value that shows how excellent or poor a health condition is based on the preferences of the nation's general population (16–18).

Various authors have reported inconsistent findings regarding the prevalence of HRQoL among people living with hypertension in different regions of Ethiopia. Therefore, the main purpose of this systematic review and meta-analysis was to estimate the pooled prevalence of HRQoL and its associated factors among people living with cardiovascular diseases in Ethiopia. In addition, despite Ethiopia's best efforts, the physical, psychological, social, and environmental aspects of quality of life among patients with cardiovascular diseases such as hypertension have not received adequate consideration. This holds true even with market-day screening for early detection, prevention, and treatment accessibility.

The quality of life among patients with cardiovascular diseases in Ethiopia has not been thoroughly examined. Therefore, this study aimed to assess the prevalence and associated factors of health-related quality of life among patients with cardiovascular diseases.

## Materials and methods

The results of this systematic review and meta-analysis were reported in accordance with the International Recommended Reporting Items for Systematic Review and Meta-analysis (PRISMA) guidelines (19) (Supplementary Material). We followed the flowchart of the PRISMA guidelines to illustrate the selection procedure, from the first identification of records to the final inclusion of studies. The review was registered in the Prospective Register of Systematic Reviews (PROSPERO) database (Reference number: CRD42024573993).

### Study setting

This systematic review and meta-analysis was conducted in Ethiopia.

### Search strategy for articles

This review focused on primary studies that assessed health-related quality of life among individuals with CVDs. A thorough search of published literature was conducted utilizing reliable databases (PubMed) and web-based search platforms (Science Direct, African Journals Online, and Google Scholar). The core search terms and phrases used were “health-related quality of life,” “quality of life,” “cardiovascular disease,” “cardiovascular disorder,” “hypertension,” “heart failure,” “determinants,” “associated factors,” and “Ethiopia.” The search was performed by two authors (WT and SW). All published articles up to 31 March 2024 were considered in this review.

### Eligibility criteria

Primary quantitative analytical studies describing the prevalence of health-related quality of life and associated factors among patients with cardiovascular diseases were included. The articles considered in this review were cross-sectional studies conducted at institutions. The review included the following types of studies: (1) published in the English language; (2) used a validated assessment method to assess and report HRQoL and associated factors in patients with CVDs; and (3) published in peer-reviewed journals. However, we excluded studies that failed to describe the desired outcome (prevalence of health-related quality of life), case reports, abstracts, or unpublished studies.

### Outcome of interest

The main outcome of interest for this review was the prevalence of health-related quality of life as measured in a primary study using a questionnaire based on the WHOQL-BREF 26-item validated checklist consisting of four domains. These domains are physical health (seven items), psychological health (six items), social relationships (three items), and environmental health (eight items). Two questions not included in any of the domains are the overall perception of HRQoL and general health perception. The scores were transformed linearly to a 0–100 scale. A higher total score denotes a higher health-related quality of life, except for Q3, Q4, and Q26, which were reverse-coded, for which a lower score denotes a higher quality of life and a higher score denotes a lower quality of life. Therefore, these three items were transformed from negatively framed to positively framed questions. The formula used to transform each domain score out of 100 is as follows: transformed domain score = (raw score − 4) × (100/16). The mean score of all items in each domain multiplied by four gives the raw data. The overall health-related quality of life was computed as the average score of four domain scores (the sum of four domain scores divided by four) (20). For the meta-analysis of secondary outcomes (factors), we extracted data on factors associated with HRQoL from primary studies. When investigating factors associated with health-related quality of life, data [adjusted odds ratio (AOR), 2 × 2 tables] from primary studies were retrieved to determine the association between risk factors and health-related quality of life.

### Article selection and data extraction

All articles retrieved from the electronic databases were imported into EndNote X7. After removing duplicates, two authors (SW and AZ) independently screened the articles, excluding the ineligible ones. Articles meeting the eligibility criteria served as data sources for the meta-analysis. The data were extracted by two authors (SW and AZ) using Microsoft Excel. The data extraction form included the following information: corresponding author name, publication year, study setting or region, study design, sample size, participants or response rate, sampling technique, tools utilized, prevalence of health-related quality of life, and associated factors. Any disagreements during data extraction were resolved through discussion with another author (WT).

### Quality assessment

The quality of the articles included in the final analysis was appraised using the Joanna Briggs Institute (JBI) quality evaluation checklist (21).

### Statistical analysis

#### Heterogeneity test

The extracted data were imported into STATA version 11 (STATA Corp., LLC) to determine the pooled prevalence of health-related quality of life. Heterogeneity among the results of the primary studies was analyzed using Cochran's *Q* test and quantified using *I*^2^ statistics. A *p*-value <0.05 indicated the presence of considerable heterogeneity. Heterogeneity was classified as low, moderate, and high when the Higgs *I*^2^ values were less than 25%, between 25% and 75%, or greater than 75%, respectively (22, 23). A random-effects model was used to estimate the prevalence of health-related quality of life among patients with cardiovascular diseases. Due to the substantial heterogeneity across the included articles, a random-effects model, namely, the DerSimonian and Laird (D + L) pooled estimate approach, was used (24). To determine the source of heterogeneity, subgroup analyses were conducted. A sensitivity analysis was also performed to explore the impact of a single study on the pooled estimate.

#### Publication bias

A visual inspection of a funnel plot was used to evaluate publication bias. A symmetrical graph approach to the origin indicated a lack of publication bias. Furthermore, Egger's regression test was performed to assess publication bias, with a *p*-value <0.05 indicating the presence of statistically significant publication bias (25).

## Results

### Study selection

**Figure 1:**
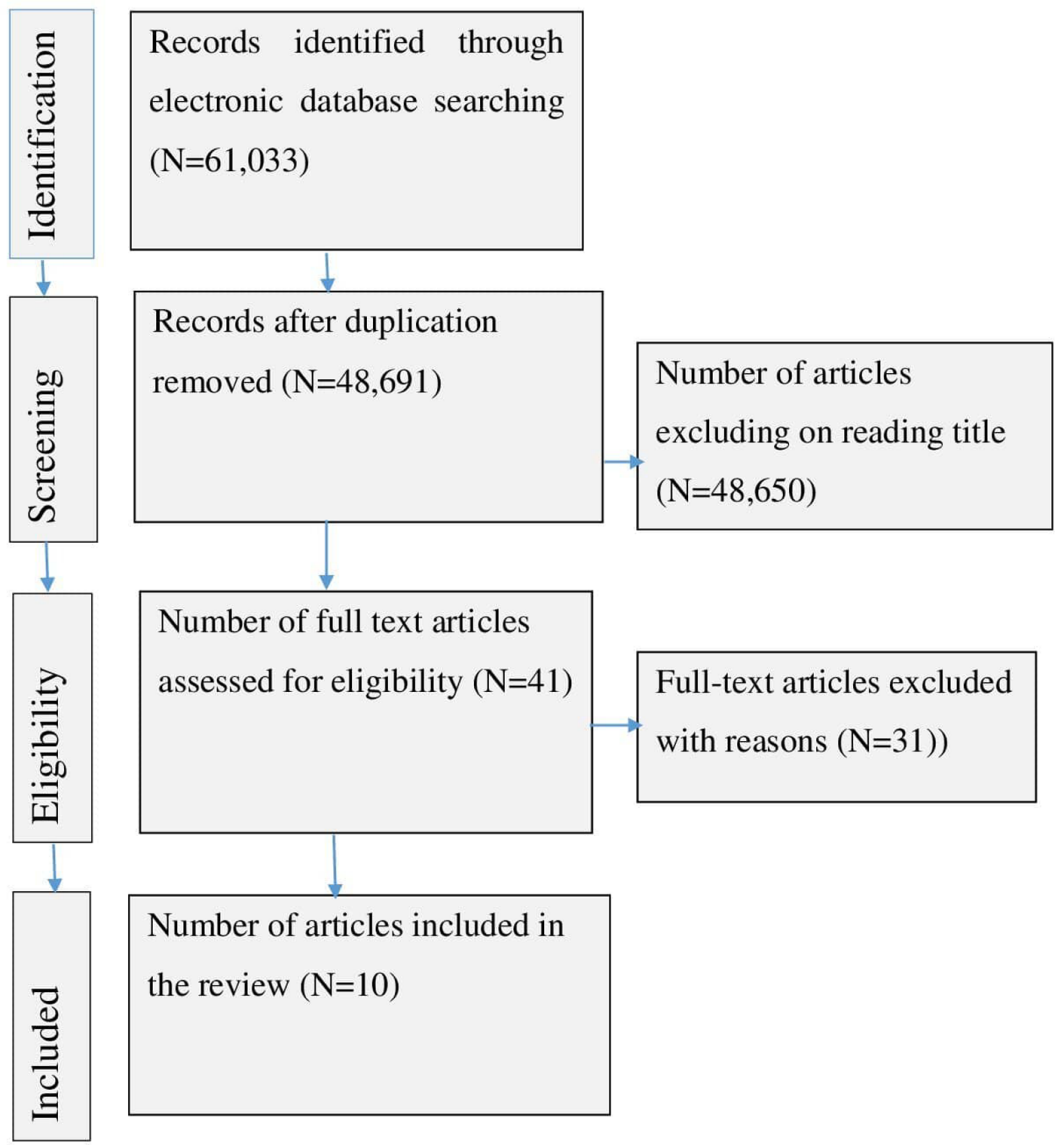
PRISMA flow diagram for a systematic review and meta-analysis of the level of health-related quality of life among people with cardiovascular disease in Ethiopia (*N* = 10).

The search yielded 61,033 articles. Of these, 58,300 were retrieved from Google Scholar, 50 were from PubMed, 1,403 were from Science Direct, and 1,280 were from African Journal Online. After reading the titles, we excluded 12,342 articles due to duplication. After screening the remaining 48,691 articles, we excluded 48,650 because they did not focus on the outcomes of interest or were not conducted on individuals with cardiovascular illness. The full texts of the remaining 41 articles were assessed for inclusion criteria, and their quality was assessed using the JBI checklist. Additional studies were excluded because it is difficult to determine the level of health-related quality of life or were not performed in Ethiopia. Finally, the analysis included 10 primary articles (Figure 1).

### Baseline study characteristics

This systematic review and meta-analysis included 10 published articles, with a total of 3,656 participants. All included articles used an institutional-based cross-sectional design. The articles were published between 2017 and 2023. Regarding the regional setting where the included studies were performed, three studies were conducted in the Amhara region (26–28), five were conducted in Addis Ababa (29–33), and the remaining were conducted in Tigray and South regions (34, 35). In terms of the sampling technique, six studies utilized non-probability sampling, while the others employed probability sampling (Table 1). All articles (10) were retrieved from Google Scholar.

**Table 1:** Baseline features of the included publications on the prevalence of health-related quality of life and associated factors in Ethiopian patients with cardiovascular illness (*n* = 10).

Primary author	Publication year	Setting	Study design	Study subjects	SS	Prevalence (%)	Sampling procedure
Adamu et al. (26)	2022	Amhara	IBCS	Hypertensive patients	376	39.4	Simple random
Shimels et al. (29)	2022	Addis Ababa	IBCS	Hypertensive patients	423	59.2	Purposive
Jufar et al. (34)	2017	Tigray	IBCS	Hypertensive patients	255	64.88	Simple random
Endalew et al. (30)	2021	Addis Ababa	IBCS	Myocardial infarction	421	29.5	Consecutive
Molla et al. (35)	2021	South	IBCS	Heart failure	388	50	Simple random
Ewnetu Tarekegn et al. (27)	2021	Amhara	IBCS	Heart failure	469	31	Consecutive
Mulugeta et al. (31)	2023	Addis Ababa	IBCS	Heart failure	383	53.26	Consecutive
Nasir et al. (32)	2023	Addis Ababa	IBCS	Rheumatic heart failure	297	44.9	Systematic random
Tito et al. (33)	2022	Addis Ababa	IBCS	CVD	360	21.6	Consecutive
Seid (28)	2020	Amhara	IBCS	Heart failure	284	51.8	Consecutive

IBCS, institution-based cross-sectional study; SS, sample size; CVD, cardiovascular diseases.

### Prevalence of poor health-related quality of life

**Figure 2:**
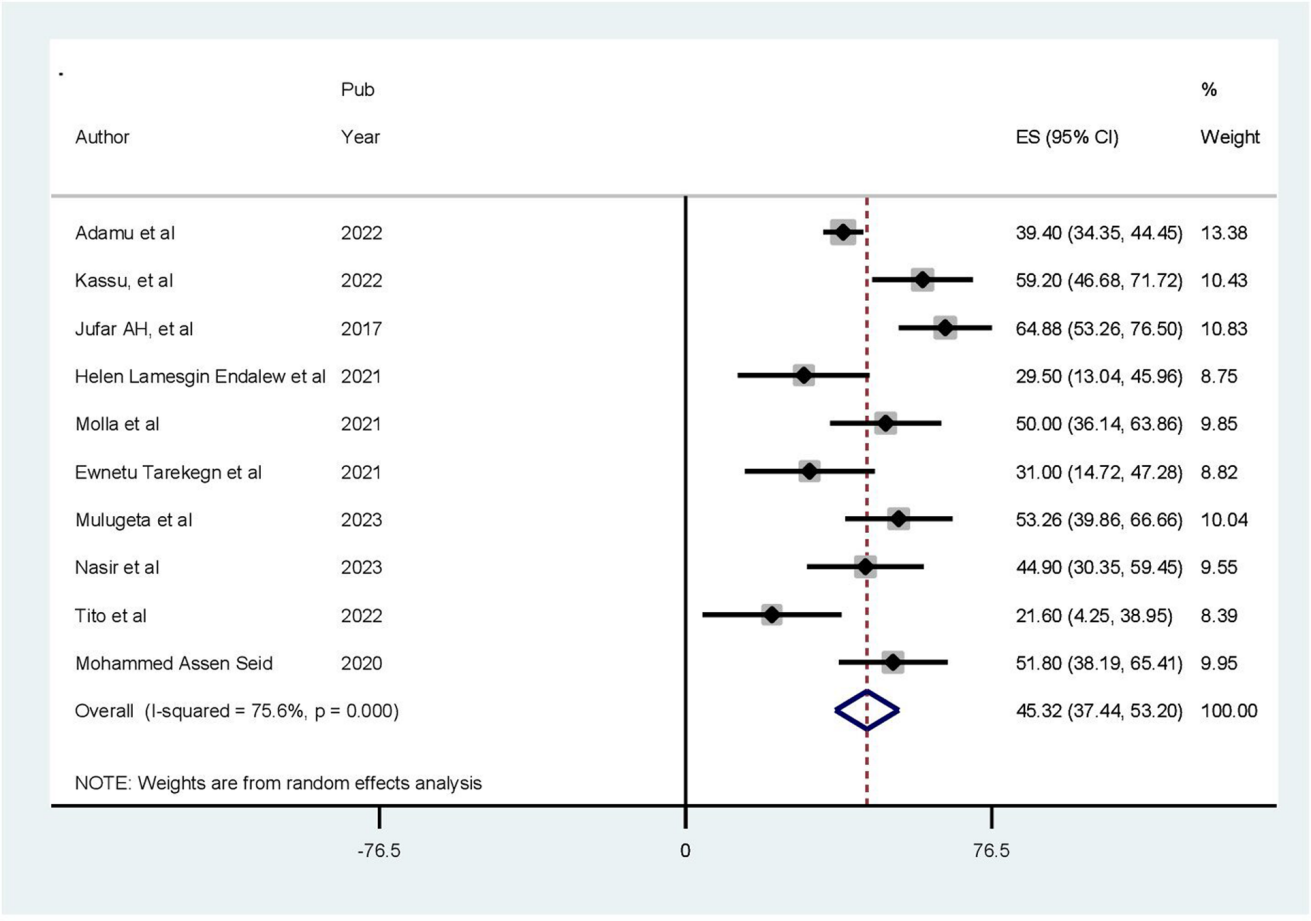
Forest plot indicating pooled prevalence of HRQoL among people with cardiovascular disease in Ethiopia (*N* = 10).

The pooled prevalence of health-related quality of life among patients with cardiovascular diseases in Ethiopia was 45.32% [95% confidence interval (CI): 37.44–53.20, *p* < 0.001]. The pooled prevalence of poor health-related quality of life was estimated using a random-effects model, specifically the D + L pooled estimate approach, due to the presence of high heterogeneity among the included studies (*I*^2^ = 75.6%, *p-*value ≤ 0.05) (Figure 2).

### Publication bias

**Figure 3:**
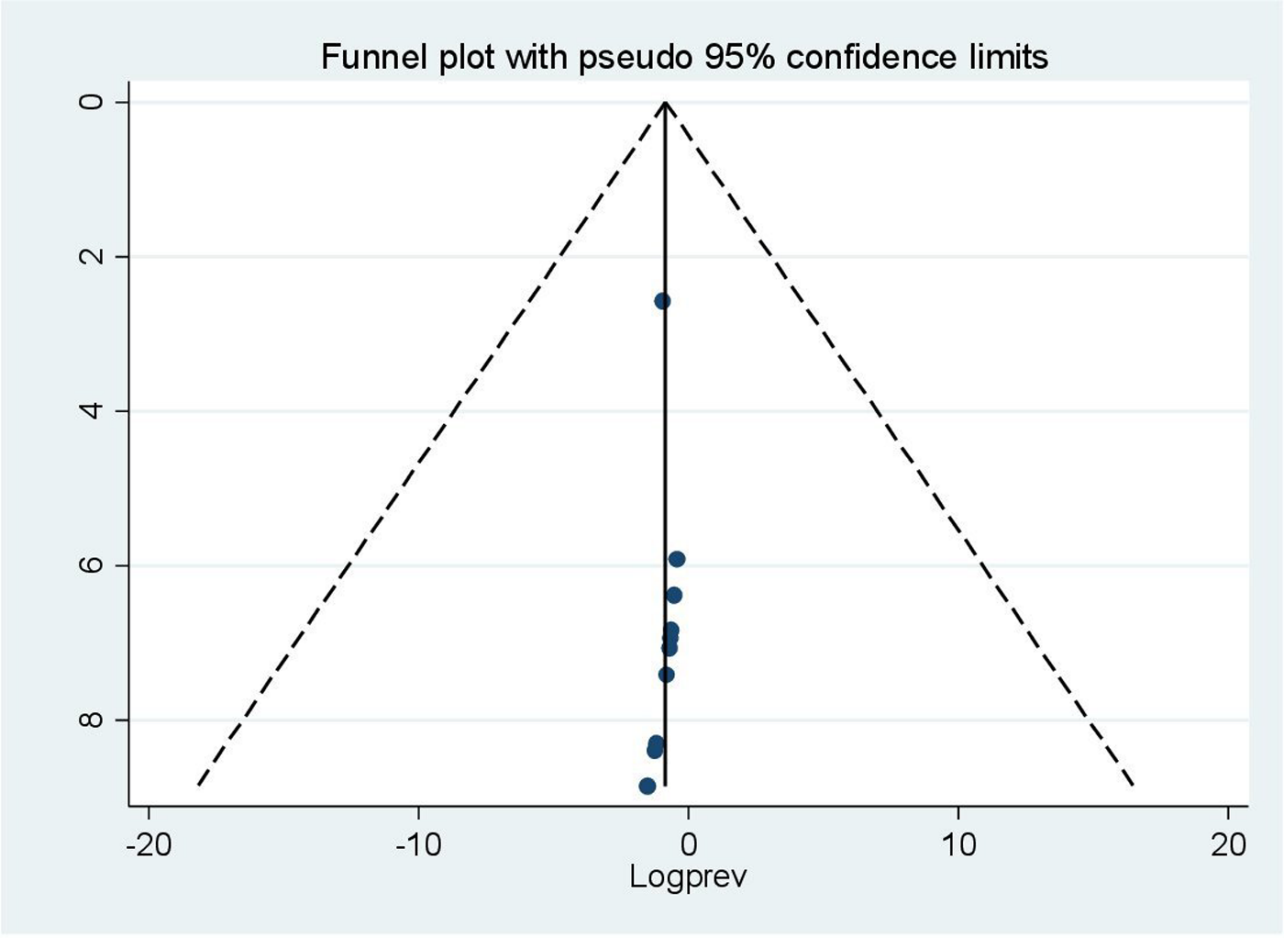
Funnel plot of health-related quality of life of patients with cardiovascular disease in Ethiopia assessed for publication bias in 10 studies, 2024.

The presence of publication bias was investigated using a funnel plot and Egger's test. The funnel plot appeared symmetrical and close to the origin (Figure 3), and Egger's test result was not significant (*p*-value = 0.885), showing that there was no publication bias among the included articles.

### Quality assessment

Based on the quality assessment, all included articles were of high quality. The quality of each of the included studies was evaluated using the JBI critical assessment checklist, which consists of nine items. The JBI quality assessment checklist consists of the following components: (1) Was the sample frame appropriate for reaching the target population? (2) Were study participants properly sampled? (3) Was the sample size appropriate? (4) Were the study participants and setting described in detail? (5) Did the data analysis provide adequate coverage of the identified sample? (6) Were appropriate procedures employed to identify the condition? (7) Was the condition assessed in a consistent, reliable manner for all participants? (8) Was there a proper statistical analysis? (9) Was the response rate adequate? For each question, a score was assigned (0 for “not reported or not acceptable” and 1 for “yes”); the scores were then summed in the range of 0–9. When the summary scores reached 0–4, 5–6, or 7–9, the studies were classified as low, medium, or high quality, respectively. The supplementary file contains detailed results from the quality assessment of the studies (Supplementary Material).

### Subgroup analysis

**Figure 4:**
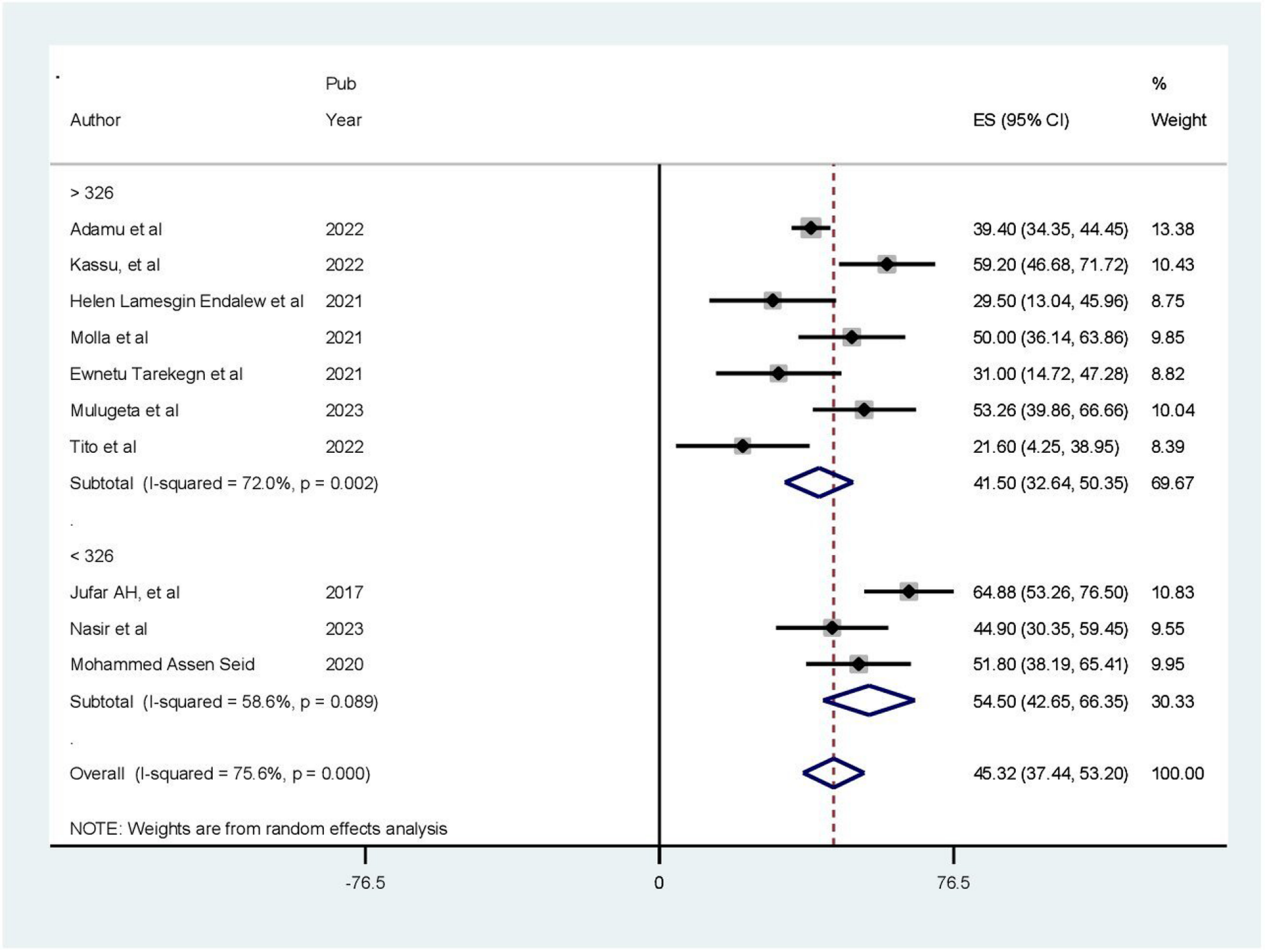
Result of subgroup analysis based on sample size (*N* = 10).

**Figure 5:**
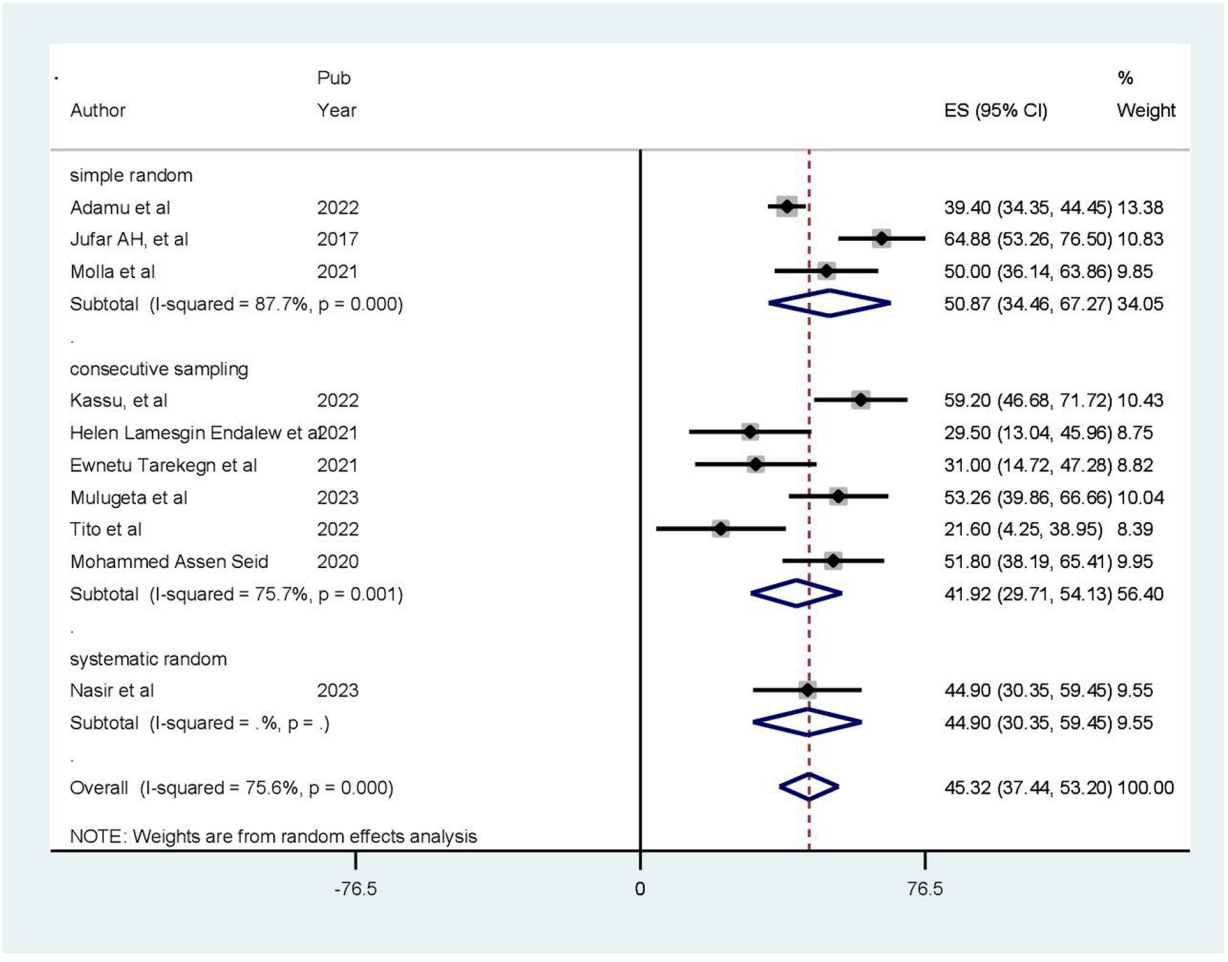
Result of subgroup analysis based on the sampling procedure (*N* = 10).

In this meta-analysis, we conducted a subgroup analysis using the sampling technique and sample size. The analysis revealed that studies with a sample size of less than 326 had the highest prevalence of poor health-related quality of life (54.50%; CI: 42.65%–66.35%) (Figure 4). The results of another subgroup analysis based on the sampling technique revealed that articles using simple random sampling had a greater prevalence of poor health-related quality of life (50.87%; CI: 34.46%–67.27%) (Figure 5).

### Sensitivity analysis

**Figure 6:**
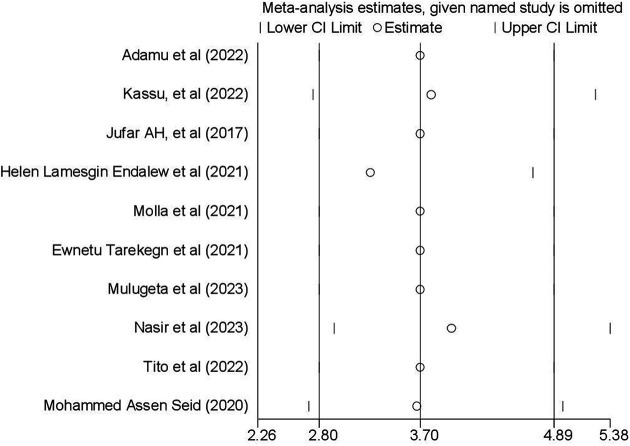
Result of sensitivity analysis to investigate influential study (*N* = 10).

To detect the influence of a single study on the pooled estimate, a sensitivity analysis was performed using a random-effects model, and the results revealed no strong evidence that any single study impacted the overall meta-analysis outcome. The graph illustrates that the estimate from a single study fell within the confidence interval of the “combined” analysis, confirming the absence of a single study effect on an overall estimate (Figure 6).

### Factors associated with health-related quality of life

**Figure 7:**
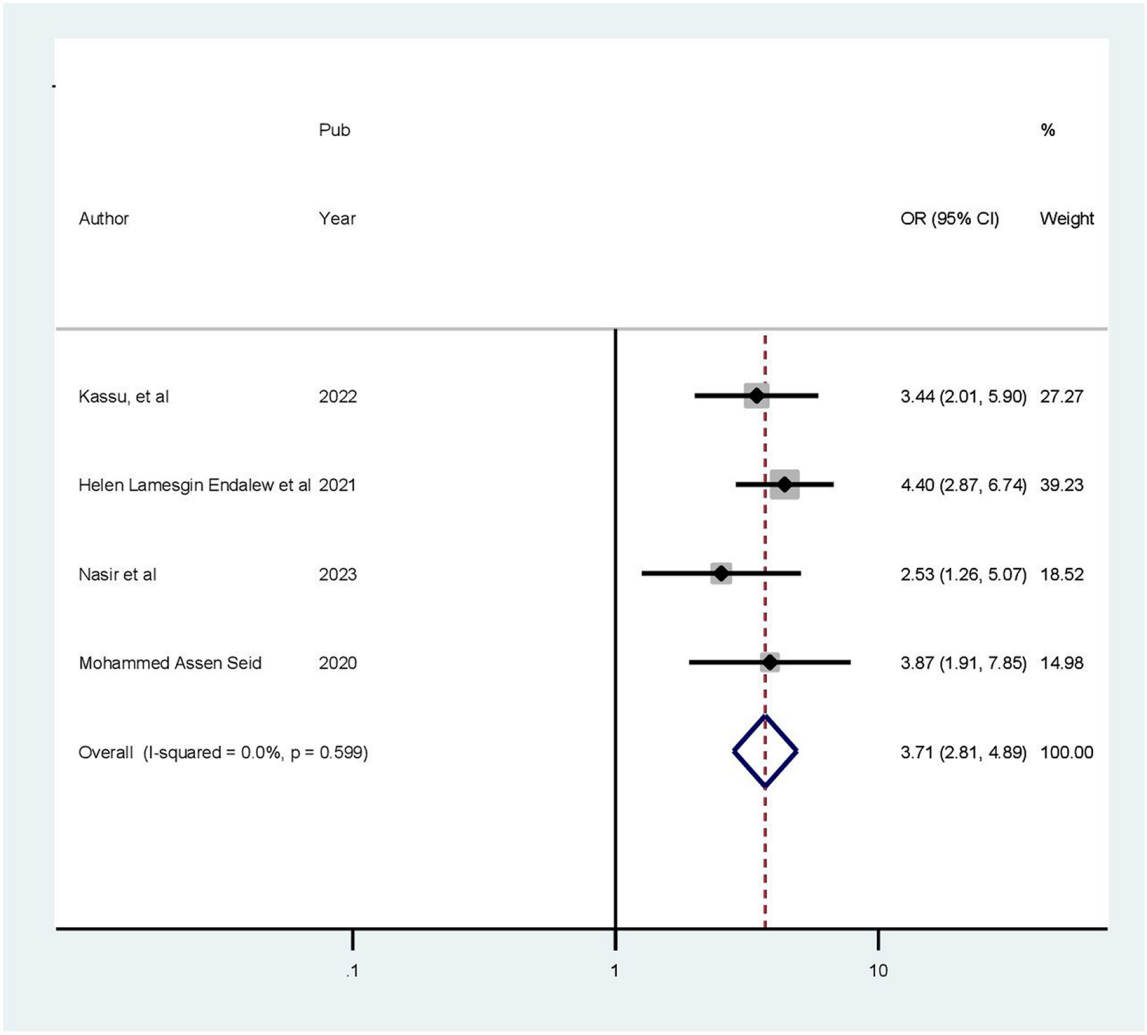
Forest plot showing the pooled odds ratio of the association between age and health-related quality of life.

**Figure 8:**
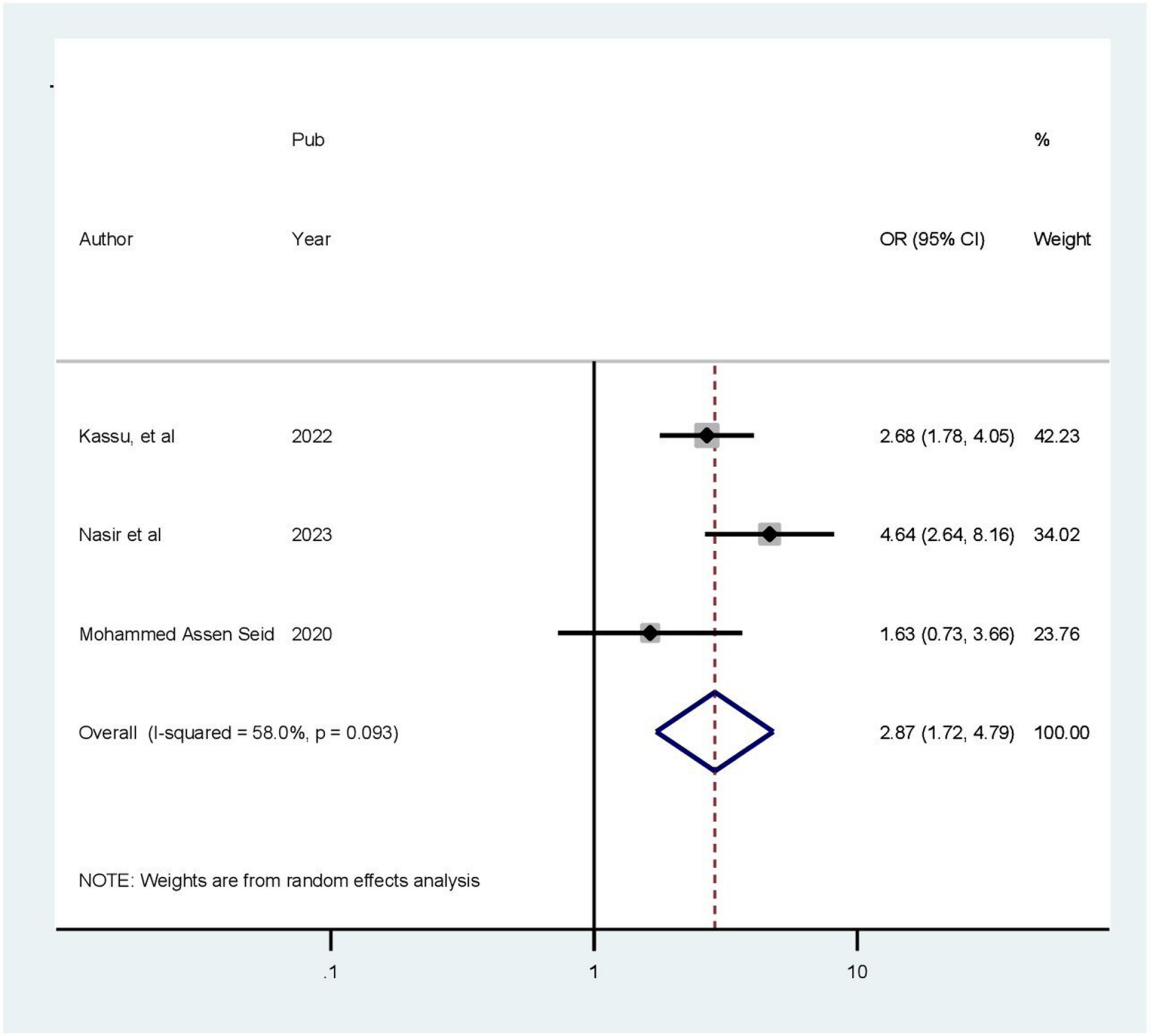
Forest plot showing the pooled odds ratio of the association between comorbidity and health-related quality of life.

**Figure 9:**
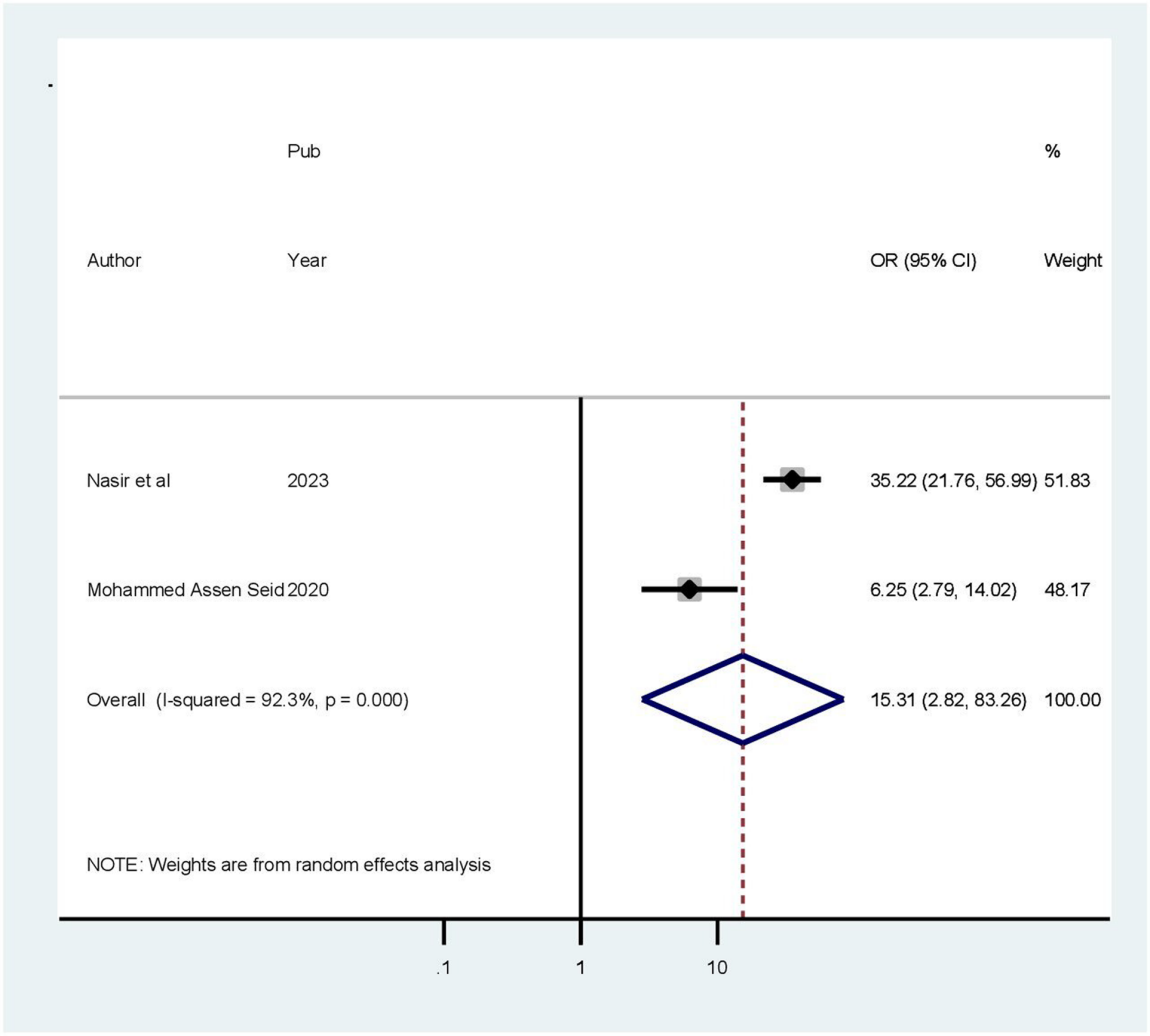
Forest plot showing the pooled odds ratio of the association between residence and health-related quality of life.

This meta-analysis included four articles investigating the association between health-related quality of life and age (28–30, 32). Patients older than 60 years were 3.71 times more likely to experience poor quality of life compared to those younger than 45 years (OR = 3.71, 95% CI: 2.81–4.89). Because a fixed effects approach was employed, the included studies revealed no heterogeneity (*I*^2^ = 0.0%, *p* = 0.383) (Figure 7). In a meta-analysis of three studies (28, 29, 32), comorbidity was found to be correlated with health-related quality of life. Accordingly, in this review, the presence of chronic comorbidities increased the odds of poor HRQoL by 87% (OR: 2.87; 95% CI: 1.72–3.4.79). The model exhibited moderate heterogeneity among the studies (*I*^2^ = 58%) (Figure 8). Two of the included studies demonstrated a statistically significant association between quality of life and rural residence (28, 32). This finding revealed that patients from rural areas had 15.31 times higher risk of experiencing poor quality of life compared to those from urban areas (OR = 15.31, 95% CI: 2.82–83.26). Using a random-effects model, the included studies exhibited heterogeneity (*I*^2^ = 92.3%) (Figure 9).

## Discussion

The objective of this study was to determine the overall prevalence of health-related quality of life among patients with cardiovascular diseases. This study revealed that patients with cardiovascular illness in Ethiopia had a pooled HRQoL prevalence of 45.32% (95% CI: 37.44–53.20, *p* < 0.001). This conclusion aligns with that of another study conducted in Iran, which indicated that the overall QOL was 39.13%, and that of a study from Ghana, which had a prevalence of 38.3% (36, 37). However, this pooled estimate is higher than that reported in studies conducted in Cameron (28.6%) and Ghana (11.39%) (38, 39). The observed gap could be attributed to variations in sociocultural elements, discrepancies in sampling techniques and sample sizes, and differences in geographic location, medical service quality, participant clinical status, study design, and study population characteristics. Another probable explanation is disparity in satisfaction with the infrastructure and healthcare services.

Patients older than 60 years were more likely to experience poor quality of life compared to those younger than 45 years. This study finding is consistent with those of other studies conducted in America, China, Nepal, and Sweden (40–43). Biologically, aging is characterized by a slow, lifelong buildup of cellular and molecular damage, eventually resulting in a reduction in physiological capabilities, an increase in susceptibility to illnesses, and an overall loss in an individual's capacity. As people age, their immune systems weaken, their musculoskeletal strength and function deteriorate, and their vision and hearing decline. As patients age, they become more susceptible to multi-morbidities, which have a greater influence on HRQoL than the combined effects of their individual diseases. As people get older, the occurrence of comorbidity becomes more common, and this has a greater impact on HRQoL than the combined impact of all of their particular diseases (44).

In this study, the presence of chronic comorbidities increased the risk of low HRQoL by 87%. This could be explained by the additional burden that comorbidities and the associated number of medications impose on a patient's health. This is because comorbidities are linked to increased healthcare needs, a greater risk of disability, greater healthcare expenses, a greater risk of financial burden, and thus social disadvantages (45).

This finding suggested that patients from rural areas have higher likelihood of experiencing a poor quality of life compared to those from urban areas. This may be due to lower literacy levels among patients with cardiovascular diseases in rural areas, making it difficult for them to adhere to self-care recommendations. In addition, the various challenges they encounter hinder their access to quality healthcare, resulting in a lower HRQoL compared to patients in urban settings (46).

### Limitations

The requirement that primary studies be published in English limited the number of studies included in this systematic review and meta-analysis. In addition, the studies evaluated were limited to certain parts of Ethiopia, which may underrepresent the rest of the country. All articles included in this study were cross-sectional with small sample sizes, which means that the outcome variable could be influenced by other confounding variables, decreasing the statistical power of the study and complicating the ability to draw causal conclusions between associated factors and patients’ quality of life.

## Conclusion and recommendations

The findings of this study reveal that a large number of patients with cardiovascular diseases in Ethiopia experience poor health-related quality of life. Furthermore, age above 60 years, the presence of comorbidities, and rural residence had substantial impacts on patients’ quality of life. As a result, this review recommends that quality-of-life evaluation be included in routine patient treatment regimens, with an emphasis on related components and domains, because it is an important tool for avoiding and treating the effects of cardiovascular diseases and significantly improving overall health. Therefore, proper attention should be given to elderly people, people with comorbidities, and rural residents.

## Data Availability

The original contributions presented in the study are included in the article/Supplementary Material, further inquiries can be directed to the corresponding author.
